# Prenatal genetic diagnosis of fetuses with dextrocardia using whole exome sequencing in a tertiary center

**DOI:** 10.1038/s41598-024-67164-w

**Published:** 2024-07-15

**Authors:** Huili Xue, Aili Yu, Lingji Chen, Qun Guo, Lin Zhang, Na lin, Xuemei Chen, Liangpu Xu, Hailong Huang

**Affiliations:** 1 Medical Genetic Diagnosis and Therapy Center, Fujian Key Laboratory for Prenatal Diagnosis and Birth Defect, Fujian Maternity and Child Health Hospital College of Clinical Medicine for Obstetrics and Gynecology and Pediatrics, Fujian Medical University, No. 18 Daoshan Road, Gulou District, Fuzhou City, 350001 Fujian Province China; 2 Reproductive Medicine Center, Fujian Maternity and Child Health Hospital College of Clinical Medicine for Obstetrics and Gynecology and Pediatrics, Fujian Medical University, No. 18 Daoshan Road, Gulou District, Fuzhou City, 350001 Fujian Province China; 3 Fujian Medical University, No. 88 Jiaotong Road, Cangshan District, Fuzhou City, 350001 Fujian Province China

**Keywords:** Dextrocardia, Prenatal diagnosis, Situs inversus totalis, Whole exome sequencing, Primary ciliary dyskinesia, Genetics, Diseases, Medical research, Molecular medicine

## Abstract

To evaluate the genetic etiology of fetal dextrocardia, associated ultrasound anomalies, and perinatal outcomes, we investigated the utility of whole exome sequencing (WES) for prenatal diagnosis of dextrocardia. Fetuses with dextrocardia were prospectively collected between January 2016 and December 2022. Trio-WES was performed on fetuses with dextrocardia, following normal karyotyping and/or chromosomal microarray analysis (CMA) results. A total of 29 fetuses with dextrocardia were collected, including 27 (93.1%) diagnosed with situs inversus totalis and 2 (6.9%) with situs inversus partialis. Cardiac malformations were present in nine cases, extra-cardiac anomalies were found in seven cases, and both cardiac and extra-cardiac malformations were identified in one case. The fetal karyotypes and CMA results of 29 cases were normal. Of the 29 cases with dextrocardia, 15 underwent WES, and the other 14 cases refused. Of the 15 cases that underwent WES, clinically relevant variants were identified in 5/15 (33.3%) cases, including the diagnostic variants *DNAH5*, *DNAH11*, *LRRC56*, *PEX10*, and *ZIC3*, which were verified by Sanger sequencing. Of the 10 cases with non-diagnostic results via WES, eight (80%) chose to continue the pregnancies. Of the 29 fetuses with dextrocardia, 10 were terminated during pregnancy, and 19 were live born. Fetal dextrocardia is often accompanied by cardiac and extra-cardiac anomalies, and fetal dextrocardia accompanied by situs inversus is associated with a high risk of primary ciliary dyskinesia. Trio-WES is recommended following normal karyotyping and CMA results because it can improve the diagnostic utility of genetic variants of fetal dextrocardia, accurately predict fetal prognosis, and guide perinatal management and the reproductive decisions of affected families.

## Introduction

Dextrocardia refers to a right-sided heart with a base–apex axis directed rightward, resulting from a variation in cardiac development. It is often accompanied by various types of cardiac defects and is prenatally correlated with high mortality due to poor prognosis^1^. Furthermore, it is difficult to treat using surgical procedures. Therefore, a definitive prenatal diagnosis of dextrocardia is vital.

The position and structure of the situs inversus totalis (SIT) are mirrored to normal; therefore, it is also called mirror-image dextrocardia^2^. The incidence of dextrocardia in live-born infants is 1 in 10,000^3^, while the incidences of 0.84, 2.3 and 0.22% were reported in prenatal series^4–6^.

To be diagnosed with a reasonably high degree of accuracy, the ultrasonographic segmental approach of fetal complex congenital heart disease^7,8^ was used: the orientation of fetal viscera should be determined first, and then the orientation of atria should be determined to diagnose the malformations. Abnormal expression of signaling molecules that maintain left–right asymmetrical differentiation leads to situs inversus (SI) and dextrocardia^9^. Dextrocardia is often accompanied by cardiovascular structural malformations, such as transposition of the great arteries, double-outlet right ventricle (DORV), tetralogy of Fallot, and ventricular septal defect (VSD).

Next-generation sequencing, particularly whole exome sequencing (WES), has been widely applied in the prenatal diagnosis of structurally abnormal fetuses^10^. Genetic analysis studies of fetal dextrocardia using WES are sparse^4,6,11^, and the diagnostic efficacy of WES in this condition has not been reported. This study aimed to explore the utility of WES for prenatal diagnosis of dextrocardia, after normal conventional karyotypes and chromosomal microarray analysis (CMA) results.

## Patients and methods

### Subjects

We prospectively recruited pregnant women undergoing invasive prenatal diagnostic procedure for fetal dextrocardia at Medical Genetic Diagnosis and Therapy Center centers in Fujian Maternity and Child Health Hospital, Fujian Medical University, China, from December 2015 to December 2022. Fetal samples were collected via invasive prenatal diagnostic procedure according different gestational weeks with informed consent. The study was approved by the Ethics Committee of the Fujian Maternity and Child Health Hospital (No.2016KYLLD01051).

### Conventional karyotyping analysis

Fetal karyotyping was performed in accordance with the standard cytogenetic protocol^12^, and karyotypes were scanned on Leica GSL120. Karyotype analysis and description were conducted following ISCN 2020^13^.

### Isolation of genomic DNA

Genomic DNA were extracted from fetal sample (30–40 mL of amniotic fluid, 15 mg of chorionc villi, or 2–5 mL umbilical cord blood) and parental blood using the QIAamp® DNA Blood Mini Kit (Qiagen Inc., Hilden, Germany) according to the manufacturer’s protocol, and the purity and concentration of genomic DNA were further determined by NanoDrop 2000 Ultramicro Spectrophotometer. maternal cell contamination was ruled out using multiplex quantitative-fluorescent polymerase chain reaction Darui kit (Darui, Guangzhou, China), which was tested on 20 short tandem repeats markers.

### Single nucleotide polymorphism array and data analysis

Submicroscopic copy number variations (CNVs) and region of homozygosity (ROH) were detected using a SNP array on a CytoScan 750 K (Affymetrix Inc., Santa Clara, CA) platform containing 200 000 SNPs and 550 000 CNVs probes, all the experimental processes of SNP array were performed as previously described^14^.

The coordinate of the chromosome was described based on the genome version hg19. The raw data were analyzed by the Affymetrix Chromosome Analysis Suite software (version 3.1.0.15). The pathogenicity of CNVs detected were classified according to the American College of Medical Genetics (ACMG) guidelines^15^. The reporting threshold was set at CNV ≥ 500 Kb and 10 Mb size for ROH in non-imprinted chromosomes or over 5 Mb for a terminal fragment ROH occurred in imprinted chromosomes 6, 7, 11, 14, 15 and 20.

### WES and bioinformatics analysis

To detect potential clinically significant SNV/indel variants, fourteen trio-WES and one only parental-WES (due to the lack of induced fetal specimen in case 5) were carried out with the informed consent of pregnant couples. After the sample genomic DNA was extracted, exon capture was conducted using Agilent Sure Select Technology (Agilent, Santa Clara, CA, USA), fragmented randomly, purified, and enriched to construct DNA libraries. Paired-end sequencing (150 bp × 2) was performed on Illumina NovaSeq 6000 (Illumina, USA) instruments following the manufacturer’s instructions (Illumina, San Diego, CA, USA).

A quality score ≥ 20 (Q20) was used to first filter out low-quality sequencing reads. The data filtering was performed further following the references^16–18^. For sequence alignment, variant calling, and annotation, the sequences were mapped to their location with the human genome reference sequence (hg19 build) using Burrows-Wheeler software (version 0.59)^19^. Briefly, to extract significant variants from the called variants, the following criteria were considered: fetal ultrasound finding and/or MRI, clinical phenotype of the proband (sibling), variant that might affect gene function, frequency of the variant in the general population, and inheritance pattern of variants in the trio analysis. For the primary findings, fetal P/LP variants and variant of uncertain significance variants (VOUS) associated with fetal phenotypes would be reported, and fetal secondary findings and incidental findings would be reported according to ACMG recommended gene list and points to consider^20,21^, respectively. In addition, we also reported carrier results (P/LP variants carried by both husband and wife that can form compound heterozygosity) for recessive diseases on pregnant couples with informed consent. All SNVs and InDels (including the minor allele frequencies < 0.01 of all known variants reported) were annotated with public population frequency databases, including NCBI dbSNP, 1000 Genomes Project, gnomAD, Exome Variant Server, as well as OMIM, SwissVar, Human Gene Mutation Database, ClinVar, and other disease databases, and only variants that were clinically or potentially relevant to the patients’ phenotype were reported. Diagnostic genetic variants were selected following autosomal recessive (AR), autosomal dominant, X-linked recessive and dominant disease inheritance models. Gene variations were interpreted according to ACMG^22^.

### Sanger sequencing

The clinical significantly candidate variants were confirmed using Sanger sequencing verification using traditional methods^23^. PCR primer sequences and protocols are available upon request. Amplified fragments were sequenced using a 96‐capillary 3730xl system (Applied Biosystems).

### Pregnancy outcome

In fetuses with dextrocardia, we collected data related to basic information, imaging findings, serological Down’s screening results, non-invasive prenatal testing results, results of standard CMA and karyotype analysis, further genetic testing results, perinatal outcomes, and follow-up information. Perinatal outcomes of fetal dextrocardia were obtained from delivery records and/or via telephone at our hospital.

### Ethics approval and consent to participate

The study complied with the principles set forth in the Declaration of Helsinki. It was approved by the Institutional Review Board of Fujian Maternal and Child Health Hospital. Written informed consent was obtained from each patient.

## Results

### Patient characteristics

After excluding fetal dextrocardia with chromosomal abnormalities and clinically significant CNVs, all 29 fetuses with dextrocardia remained non-diagnostic. Gestational weeks at diagnosis of dextrocardia ranged from 13^+1^ weeks to 27^+2^ weeks, the median gestational weeks were 20 weeks.

### Genetic testing results

**Figure 1:**
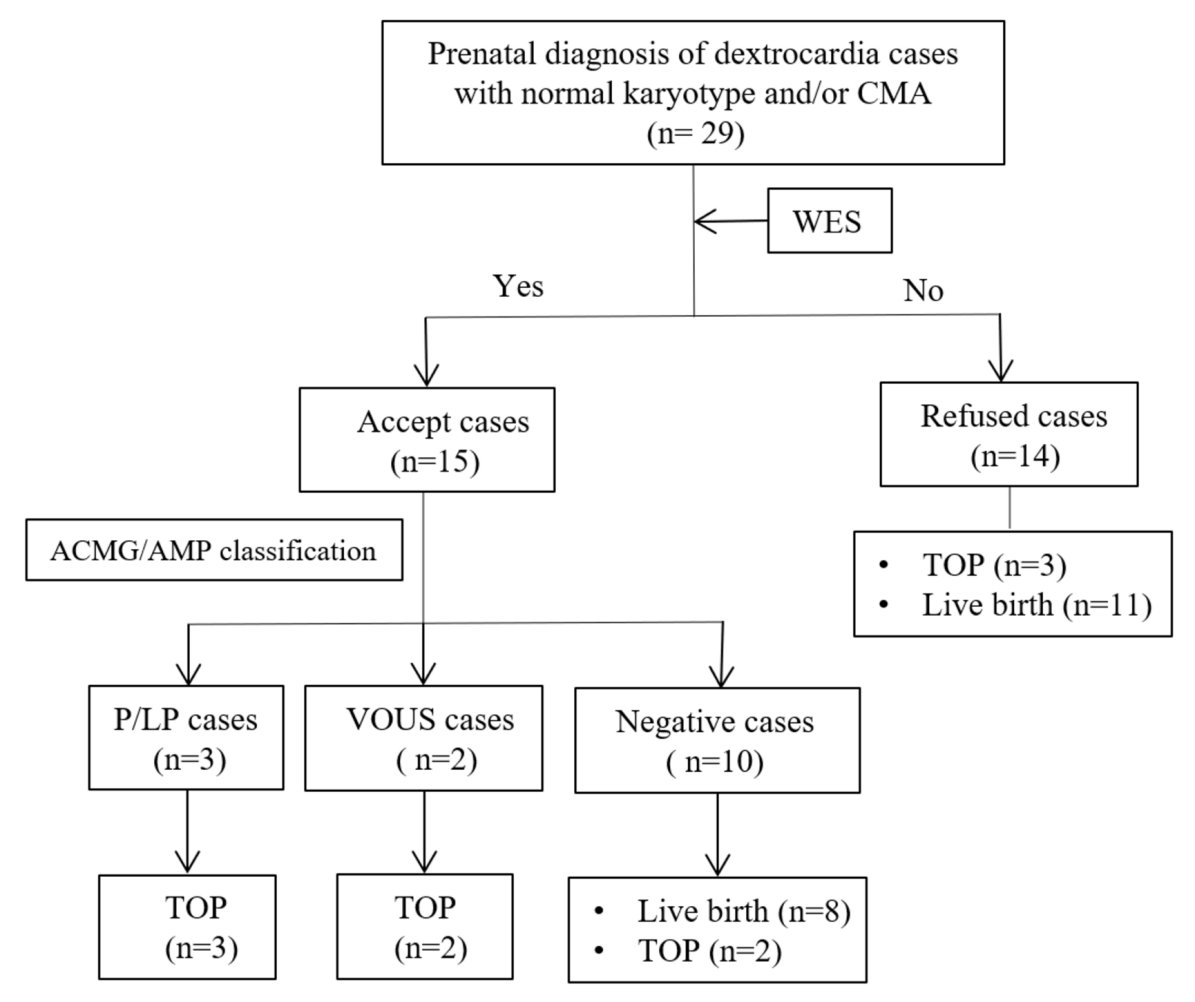
The flow chart of analysis in our cohort.

All 29 fetuses with dextrocardia had normal karyotypes and CMA results, of which 14 required further trio-WES testing and 1 case performed WES testing of the pregnant couple due to lack of induced fetal samples. A flowchart of our cohort analysis is shown in Fig. 1. Overall, four cases with clinically relevant variants were detected in 14 fetuses, and one case with diagnostic variant was identified in the affected parent (Case 5), associated with diverse types of primary ciliary dyskinesia (PCD), peroxisome biogenesis disorder 6A (Zellweger)/6B, and X-linked recessive non-syndromic congenital heart defects 1. The diagnostic variants identified were involved in *DNAH5*, *DNAH11*, *LRRC56*, *POEX10*, and *ZIC3* (Table 1).

**Table 1:** Genetic testing results detected by WES and the outcomes of 15 fetal dextrocardia with normal karyotyping and CMA results.

Case ID	Fetal ultrasound finding	Ref Gene/Transcripts	Variant/Consequence/GRCh37 dbSNP	Novel/Previously reported (PMID)	Origin	ACMG classification	Zygosity	Consanguinity status	Associated disorder, OMIM, Inheritance model	Pregnancy outcomes
Clinically relevant variants identified										
1	SIT, mirror-image dextrocardia, bilateral ventriculomegaly	*DNAH11* NM_001277115.2	c.10036C > T (p.Arg3346*) Nonsense variant Chr7:21,840,764 rs773851007	PMID:26,909,801	Mat/	Pathogenic (PVS1, PM3, PM2_Supporting)	Het	No	PCD type 7, with or without situs inversus, 611,884 AR	TOP
			c.5822G > C (p.Trp1941Ser) Missense variant Chr7:21,727,043 rs779535145	Novel	Pat	VOUS (PM3, PP3, PM2_Supporting)	Het			
2	SIT, mirror-image dextrocardia, the spinal conus is located at the lower margin level of L3	*LRRC56* NM_198075.4	c.760G > T (p.Glu254*) Nonsense variant Chr11:551,266 rs372959912	PMID:30,388,400	Pat	Pathogenic (PVS1 + PM3 + PM2_Supporting)	Het	No	PCD type 39, 618,254 AR	TOP
			c.1053dupC (p.Glu352Argfs*2) Frameshift variant Chr11:552,098 rs761092893	Novel	Mat	Pathogenic (PVS1, PM3, PM2_Supporting)	Het			
3	SIT, mirror-image dextrocardia, VSD, recurrent adverse pregnancy history twice	*DNAH5* NM_001369.3	c.6304C > T (p.Arg2102Cys) Missense variant Chr5:13,829,759 rs767019228	PMID: 32,502,479, 33,974,255, 34,556,108	Mat	LP (PM3_Strong, PM2_Supporting, PP1)	Het	No	PCD type 3, with or without situs inversus AR	TOP
			c.4355 + 5G > A Splicing region variant Chr5:13,865,772 rs774366812	PMID:36,003,331	Pat	LP (PM3_Strong, PM2_Supporting, PP1, PP3)	Het			
4	SIT, mirror-image dextrocardia, thicky NT, DORV, pulmonary stenosis, absence of pulmonary valve, recurrent adverse pregnancy history twice	*DNAH11* NM_001277115.2	c.1778G > A (p.Ser593Asn) Missense variant Chr7:21,627,749 rs754774362	Novel	Pat	VOUS (PM2_Supporting)	Het	No	PCD type 7, with or without situs inversus, 611,884 AR	TOP
			c.11352C > A (p.Phe3784Leu) Missense variant Chr7:21,901,620 rs553539918	Novel	Pat	VOUS (PM2_Supporting)	Het			There was a 2 cm hematoma at the back of the fetus' head
		*PEX10* NM_002617.4	c.814_815del (p.Leu272Valfs*66) Frameshift variant Chr1:2,338,020 rs61752093	PMID: 12,794,690, 17,041,890, 19,142,205, 21,031,596	Pat	Pathogenic (PVS1, PS4, PM2_Supporting	Het		Peroxisome biogenesis disorder 6A/6B, 614,870/614,871 AR	
			c.928 T > C (p.Cys310Arg) Missense variant Chr1:2,337,258 rs753384584	Novel	Mat	VOUS (PM3, PP3, PM2_Supporting)	Het			
5*	Persistent truncus arteriosus, dextrocardia, history of adverse pregnancy	*ZIC3* NM_003413	c.100_122del (p.Met35Leufs*87) Frameshift variant ChrX:136,648,950	Novel	Mat	LP (PVS1, PM2)	hemi	No	X-linked nonsyndromic congenital heart defects 1 type, 306,955 XLR	He died three months after birth
Negative results identified										
6	Dextrocardia, VSD, hepatic hypoplasia of inferior vena cava	*DNAH11* NM_001277115.2	c.3426-1G > A Splicing region variant Chr7:21,641,013 rs774855011	Novel	Mat	Pathogenic (PVS1,PM3,PM2_Supporting)	Het	No	PCD type 7, with or without situs inversus, 611,884 AR	LB
7	SIT, mirror-image dextrocardia, enhanced intestinal tube echo	NEG	−		−	−	−	No	−	LB
8	Abdominal visceral situs inversus, DORV, TGA, PLSVA (Taussing-Bing syndrome), mitral regurgitation	NEG	−		−	−	−	No	−	TOP
9	SIT, mirror-image dextrocardia	*DNAAF5* NM_017802.4	c.2246 T > C (p.Leu749Ser) Missense variant Chr7:819,596	Novel	Pat	VOUS (PM2_Supporting, PP3)	Het	No	PCD type 18, 614,874 AR	LB
10	SIT, mirror-image dextrocardia	*DNAAF2* NM_018139.3	c.736 T > G (p.Leu246Val) Missense variant Chr14:50,101,132 rs751253892	Novel	Pat	VOUS (PM2_Supporting)	Het	No	PCD type 10, 612,518 AR	LB
11	SIT, mirror-image dextrocardia	*DNAH11* NM_001277115.2	c.4307G > A (p.Arg1436Gln) Missense variant Chr7:21,658,770 rs766184035	Novel	Mat	VOUS (PM2_Supporting)	Het	No	PCD type 7, with or without situs inversus, 611,884 AR	LB
		*TTC12* NM_017868.4	c.1420 T > C (p.Phe474Leu) Missense variant Chr11:113,222,903 rs782122045	Novel	Mat	VOUS (PM2_Supporting)	Het		PCD type 45, 618,801 AR	
		*STK36* NM_015690.5	c.2315G > A (p.Arg772Gln) Missense variant Chr2:219,558,685 rs199736780	Novel	Mat	VOUS (PM2_Supporting)	Het		?PCD type 46, 619,436 AR	
12	Thoracic organs situs inversus, heterotaxy, PRUV	NEG	−		−	−	−	No	−	LB
13	SIT, mirror-image dextrocardia, left intra-cardiac echogenic foci	*DNAH1* NM_015512.5	c.1970C > G p.Pro657Arg Missense variant Chr3:52,381,854	Novel	Mat	VOUS (PM2_Supporting)	Het	No	PCD type 37, Spermatogenic failure 18 AR	TOP
14	SIT, mirror-image dextrocardia,	*HYDIN* NM_001270974.2	c.12127G > A (p.Glu4043Lys) Missense variant chr16:70,893,973 rs759245607	Novel	Mat	VOUS (PM2_Supporting)	Het	No	PCD type 5 AR	LB
		*CCDC39* NM_181426.2	c.1692A > C (p.Leu564Phe) Missense variant chr3:180,359,963	Novel	Mat	VOUS (PM2_Supporting)	Het		PCD type 14 AR	
15	SIT, mirror-image dextrocardia, tricuspid atresia, DORV	*RSPH1* NM_080860.4	c.119A > G (p.Asn40Ser) Missense variant Chr21:43,913,125 rs751918978	Novel	Mat	VOUS (PM2_Supporting)	Het	No	PCD type 24, 615,481 AR	TOP

*Since the male fetus had been induced labor in another hospital and no biological samples remained, WES examination of both the husband and wife indicated that the pregnant woman harbored a heterozygous mutation, NM_003413: c.100_122del (p.M35Lfs*87), in *ZIC3* (300,265). Considering that the male fetus of the previous pregnancy showed persistent arterial trunk and died at 6 months after birth, we speculated that the fetus was highly likely to have the hemizygous mutation, NM_003413: c.100_122del (p.M35Lfs*87), in *ZIC3* (300265).

*DORV* double outlet right ventricle, *ECD* endocardial cushion defect, *Hem* hemizygous, *Het* heterozygous, *Hom* homozygous, *LAI* left atrial isomerism, *Mat* maternal, *NEG* negative, *NT* nuchal translucency, *Pat* paternal, *PCD* primary ciliary dyskinesia, *PLSVA* persistent left superior vena cava, *PRUV* persistent right umbilical vein, *SIT* situs inversus totails, *TGA* transposition of great artery, *VSD* ventricular septal defect.

#### Cases with clinically relevant variants

**Figure 2:**
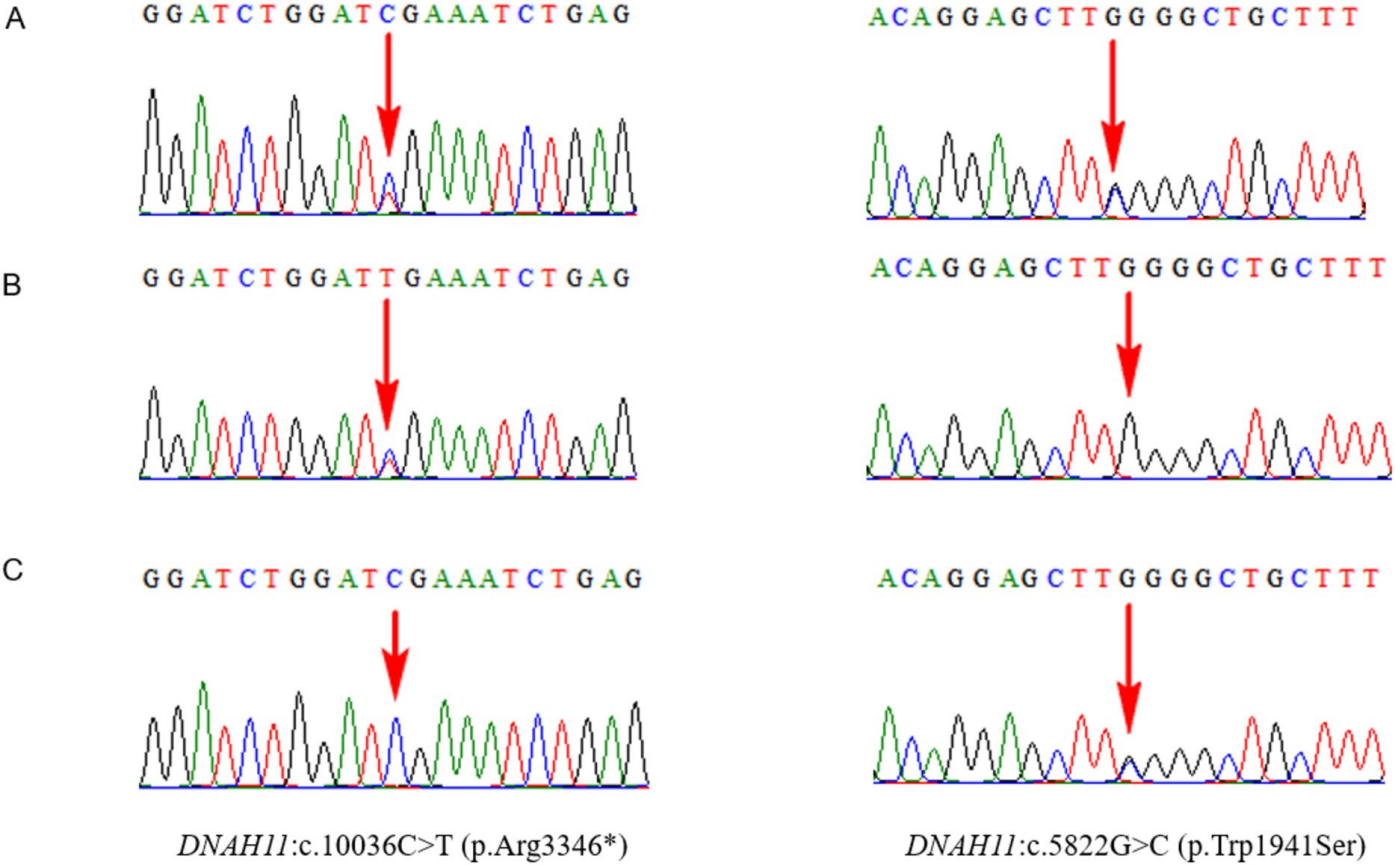
Trio whole-exome sequencing results for case 1. The left panel showing the *DNAH11*: c.10036C > T (p.Arg3346*) variant which the region indicated by the red arrow. The right panel showing the *DNAH11*: c.5822G > C (p.Trp1941Ser) variant which the region indicated by the red arrow. (**A**) Sanger sequencing analysis of the fetus. (**B**) Sanger sequencing analysis of the mother. (**C**) Sanger sequencing analysis of the father.

In Case 1, the fetus of a Chinese couple presented with SIT, mirror-image dextrocardia, and bilateral ventriculomegaly. Trio-WES revealed compound heterozygous c.10036C > T (p.Arg3346*) and c.5822G > C (p.Trp1941Ser) in *DNAH11*. Compound heterozygous variants in *DNAH11* are known to cause PCD type 7, with or without situs inversus (SI) (OMIM 611,884). Variants are extremely rare in the population, according to gnomAD. The frequency of this nonsense variant, c.10036C > T (p.R3346*), is low in the normal reference population gene database (1000 Genome; gnomAD:0.00003756). It was interpreted as pathogenic according to ACMG/AMP guidelines (PVS1, PM3, PM2_Supporting), resulting in the premature termination of the codon and is expected to result in truncation of the *DNAH11* protein; a homozygous p.Arg3346* variant in *DNAH11* has been reported in two patients from one closely related family^24^. The frequency of the missense variant c.5822G > C (p.Trp1941Ser) in *DNAH11* was low in the normal reference population database [1000 Genome:.; gnomAD:0.00001198] and was interpreted as VOUS (PP3, PM2_Supporting, PP3), according to the ACMG guidelines. However, multiple computer-aided analyses predicted that the variation was more likely to affect protein structure/function (REVEL_score:0.652; GERP++_RS: 5.75; dbscSNV_ADA:.; dbscSNV_RF:.); thus, both variants supported the clinical phenotypes of the fetus^25^ (Fig. 2).

**Figure 3:**
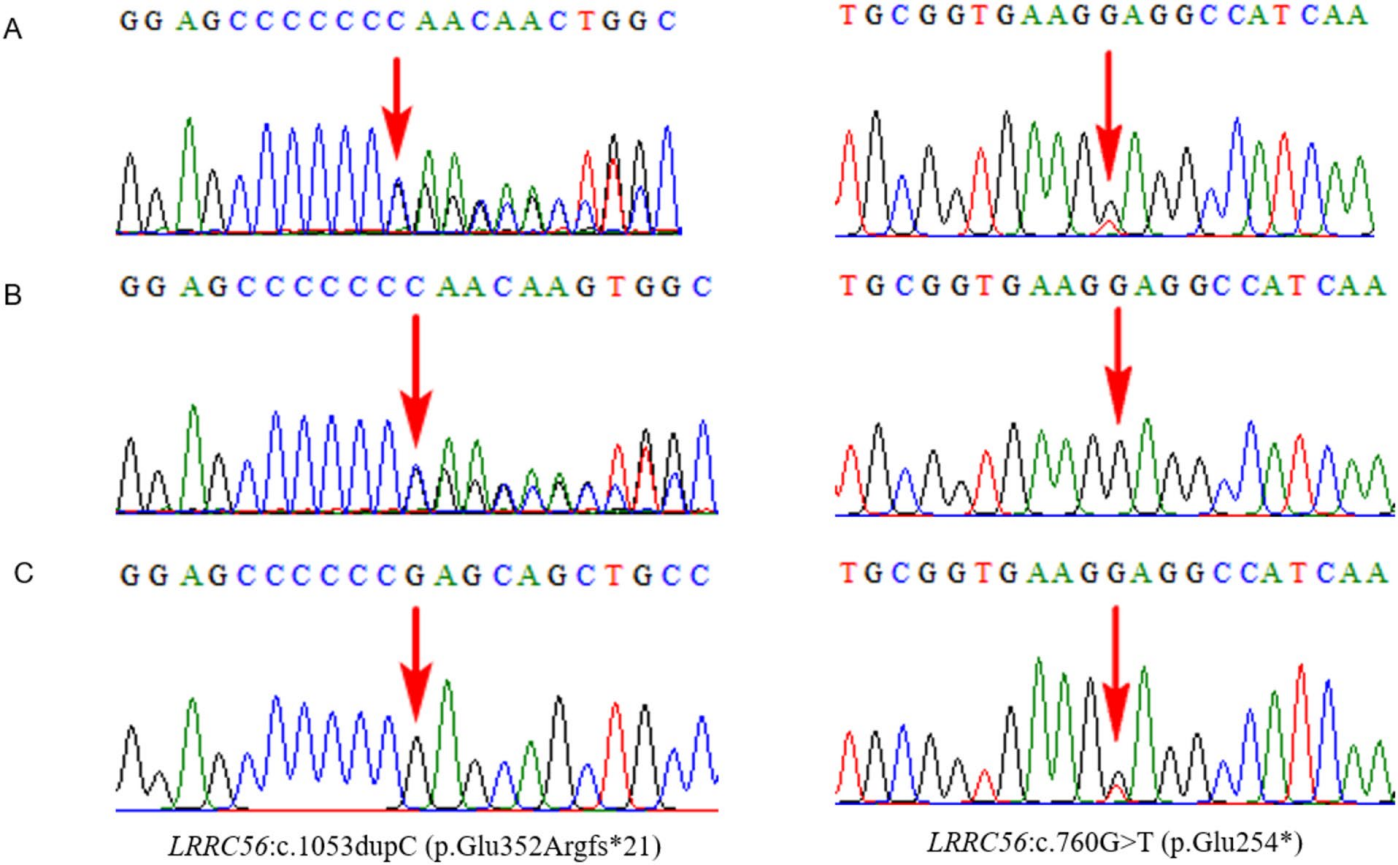
Trio whole-exome sequencing results for case 2. The left panel showing the *LRRC56*: c.1053dupC (p.Glu352Argfs*21) variant which the region indicated by the red arrow. The right panel showing the *LRRC56*: c.760G > T (p.Glu254*) variant which the region indicated by the red arrow. (**A**) Sanger sequencing analysis of the fetus. (**B**) Sanger sequencing analysis of the mother. (**C**) Sanger sequencing analysis of the father.

The fetus of a Chinese couple in Case 2 presented with SIT and mirror-image dextrocardia; the spinal conus was located at the lower margin of the third lumbar vertebra. A compound heterozygous variant, c.760G > T (p.Glu254*) and c.1053dupC (p.Glu352Argfs*21), in *LRRC56* was identified by trio-WES, both of which were interpreted as pathogenic according to the ACMG/AMP guidelines (PVS1, PM3, PM2_Supporting), causing PCD type 39 (OMIM 618254). The biallelic variants c.760G > T (p.Glu254*) and c.326 + 1G > A have been reported in three unrelated families with PCD type 39^26^. In addition, a novel heterozygous frameshift variant, c.1053dupC (p.Glu352Argfs*21), in the trans position of *LRRC56* was detected, and both biallelic variants were in accordance with the presentation of the fetus (Fig. 3). To our knowledge, this is the first report on spinal anomalies in a prenatal case of *LRRC56* variant.

**Figure 4:**
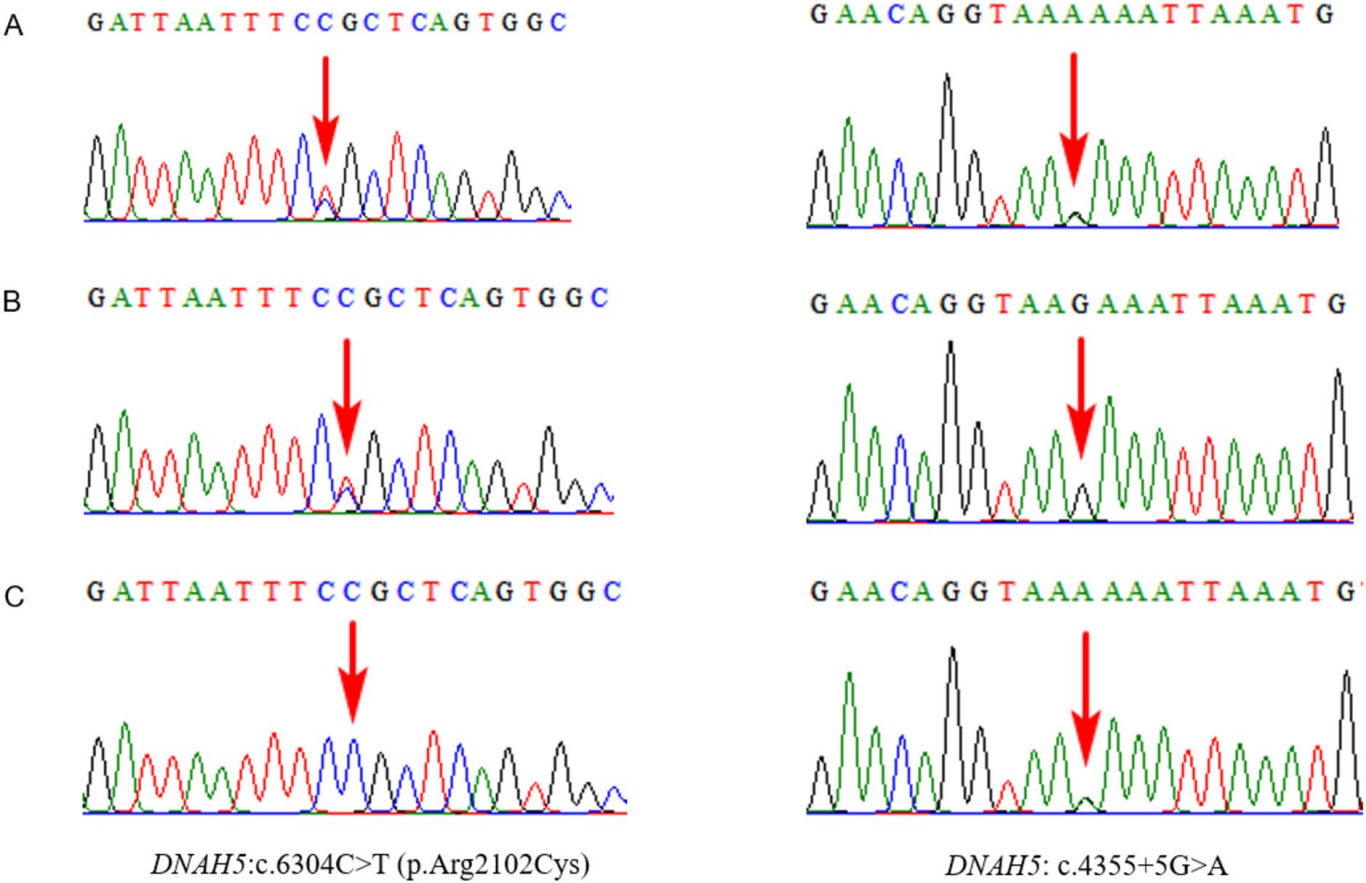
Trio whole-exome sequencing results for case 3. The left panel showing the *DNAH5*: c.6304C > T (p.Arg2102Cys) variant which the region indicated by the red arrow. The right panel showing the *DNAH5*: c.4355 + 5G > A variant which the region indicated by the red arrow. (**A**) Sanger sequencing analysis of the fetus. (**B**) Sanger sequencing analysis of the mother. (**C**) Sanger sequencing analysis of the father.

Case 3 was the third pregnancy in a Chinese couple complicated by recurrent fetal SIT, mirror-image dextrocardia, VSD, and a right choroid plexus cyst. The first pregnancy was complicated by fetal VSD, aortic straddle, pulmonary stenosis, aberrant right subclavian artery, tricuspid regurgitation, femur length < 4.4 standard deviation (SD) from the mean, humerus length < 3.5 SD from the mean, skeletal anomalies, increased nuchal fold, ventriculomegaly, and unclear gallbladder, and the fetus died in utero. The parents refused to undergo genetic testing. In the second pregnancy, the fetus presented with situs inversus, a cardiac malformation (levoversion of heart, DORV, pulmonary stenosis, and right aortic arch), increased nuchal translucency, and a cervical cyst. Trio-WES revealed that the fetus carried compound heterozygous variants, c.6304C > T and c.4355 + 5G > A in *DNAH5*, inherited from the mother and father, respectively (Fig. 4). Both were interpreted as VOUS, according to the ACMG guidelines. The fetus was born in 2020 and died days after birth. Currently, this is the third pregnancy. The fetus was again affected by SI and dextrocardia. Trio-WES detected compound heterozygous variants c.6304C > T (maternal) and c.4355 + 5G > A (paternal) in *DNAH5* again. Biallelic loss-of-function variants in *DNAH5* are known to result in PCD type 3, with or without SI (OMIM 608,644). Both variants are extremely rare in the population, according to gnomAD. The c.6304C > T (p.Arg2102Cys) variant of *DNAH5* has previously been reported in patients with PCD type 3 and SI^27,28^. This variant, c.4355 + 5G, has been previously detected in 2 patients with PCD to form a complex heterozygous mutation with the c.10438G > T(p.Glu3480Ter) variant^29^. c.6304C > T and c.4355 + 5G > A were both classified as likely pathogenic according to ACMG guidelines. Considering the recurrent abnormal fetal phenotypes and genetic testing results, the couple opted to terminate the pregnancy.

**Figure 5:**
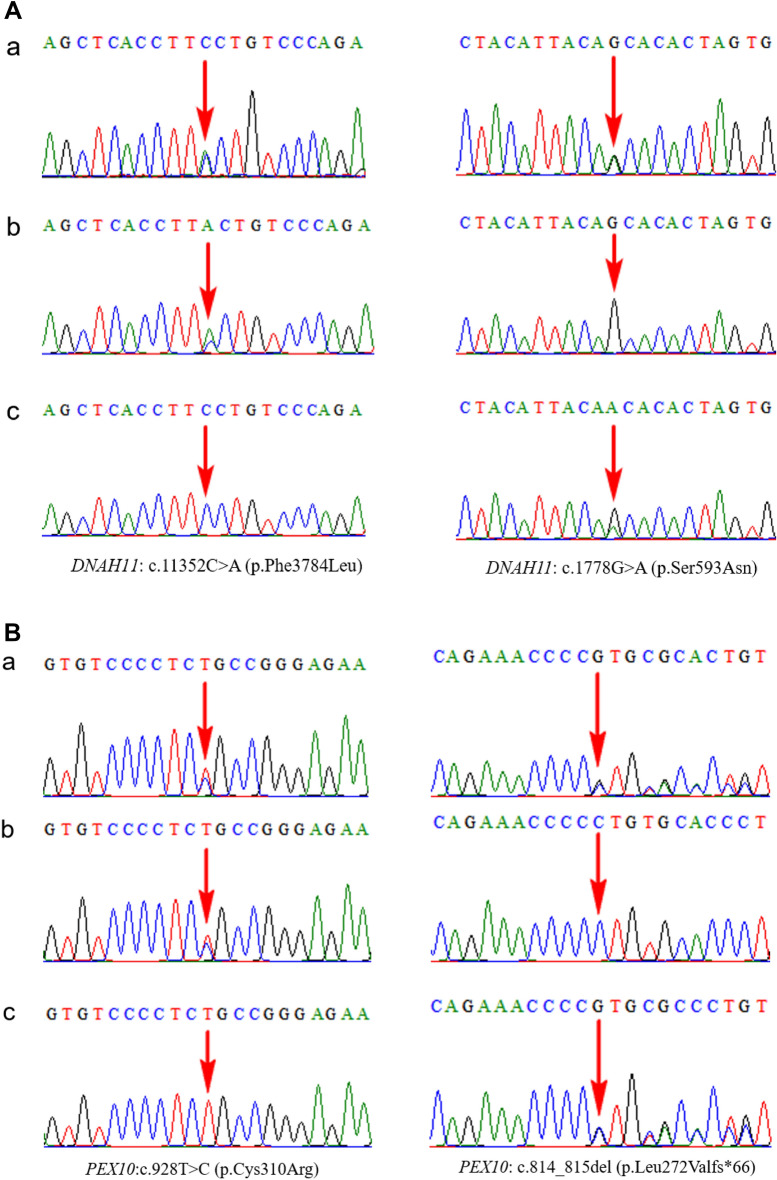
Trio whole-exome sequencing results for case 4. (**A**) The left panel showing the *DNAH11*: c.11352C > A (p.Phe3784Leu) variant which the region indicated by the red arrow. The right panel showing the *DNAH11*: c.1778G > A (p.Ser593Asn) variant which the region indicated by the red arrow. (**a**) Sanger sequencing analysis of the fetus. (**b**) Sanger sequencing analysis of the mother. (**c**) Sanger sequencing analysis of the father. (**B**) The left panel showing the *PEX10*: c.928 T > C (p.Cys310Arg) variant which the region indicated by the red arrow. The right panel showing the *PEX10*: c.814_815del (p.Leu272Valfs*66) variant which the region indicated by the red arrow. (**a**) Sanger sequencing analysis of the fetus. (**b**) Sanger sequencing analysis of the mother. (**c**) Sanger sequencing analysis of the father.

Case 4 was the third pregnancy of a Chinese couple and the fetus was affected by situs inversus, mirror-image dextrocardia, thick nuchal translucency (NT), DORV, pulmonary stenosis, and the absence of a pulmonary valve. In the first pregnancy, the fetus presented with increased NT (3.8 mm), VSD, the cerebellar inferior vermis was suspiciously absent, at 27 weeks of gestation, the fetal cranial magnetic resonance imaging (MRI) showed that the biparietal diameter was smaller than the gestational age, bilateral ventriculomegaly, the corpus callosum was thinner, the posterior fossa cistern was inhomogeneously enlarged, and the lower part of the cerebellar vermis was smaller than the gestational age; Dandy-Walker malformation could not be excluded. The parents chose to terminate the pregnancy and refused genetic testing of the fetus. In their second pregnancy, conceiving naturally, the fetus was affected by increased NT (2.8 mm), dextrocardia, and skin edema throughout the body, died in utero, and the CMA result of the fetus was normal. Trio-WES revealed that the fetus carried a compound heterozygous variant, c.1778G > A (p.Ser593Asn) and c.11352C > A (p.Phe3784Leu), in *DNAH11,* associated with PCD type 7, with or without SI (OMIM 611884)^30^, as well as another compound heterozygous variant, c.928 T > C (p.Cys310Arg) and c.814_815del (p.Leu272Valfs*66), in *PEX10,* related to peroxisome biogenesis disorder 6A (Zellweger) (OMIM 614870) and 6 B (OMIM 614871), inherited from the mother and father, respectively. All of these were interpreted as VOUS according to the ACMG guidelines, except for variant c.814_815del, which was pathogenic^31–34^. Finally, the fetus was terminated. The current pregnancy is the third; however, the fetus was again affected by dextrocardia and cardiac defects. The result of the trio-WES was the same as that of the previous fetus (Fig. 5). Genetic variants were highly correlated with the clinical manifestations in the three fetuses. Finally, the fetus was terminated.

**Figure 6:**
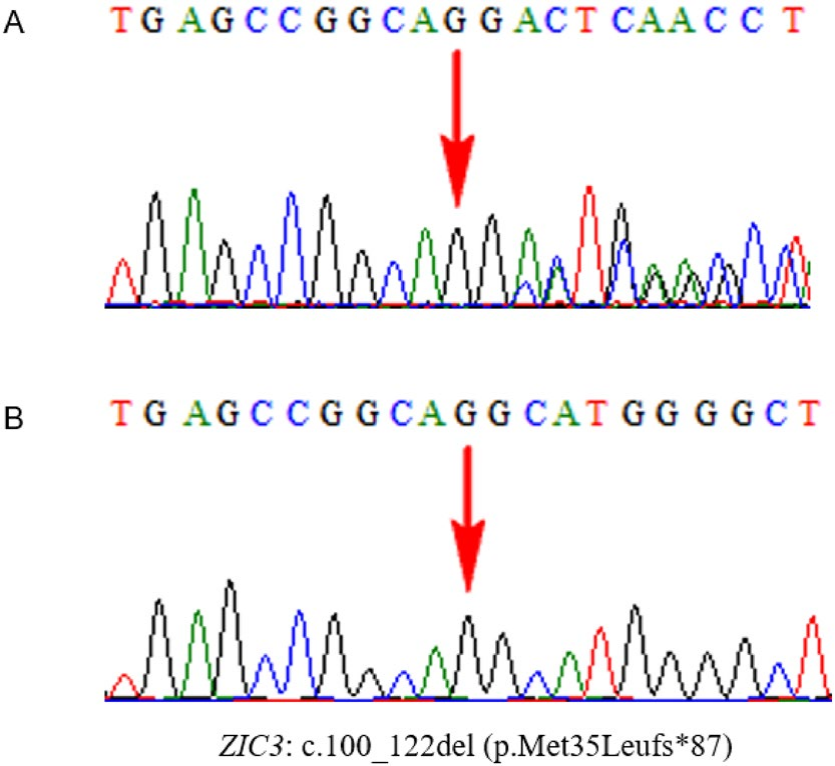
Whole-exome sequencing and Sanger sequencing results of the pregnant couple for case 5. (**A**) The pregnant mother harbored a heterozygous variant, NM_003413: c.100_122del (p.Met35Leufs*87), in *ZIC3* on chromosome X. (**B**) Wild-type of the father.

Case 5 was the third pregnancy of a Chinese couple who presented with recurrent fetal truncus arteriosus and dextrocardia. Their first pregnancy was complicated by fetal truncus arteriosus, and the male fetus died 6 months after birth. In the second pregnancy, the male fetus was again affected by truncus arteriosus, and the pregnancy was terminated. The current pregnancy was the third, and the male fetus had truncus arteriosus as well as dextrocardia. The parents terminated the pregnancy in another local hospital and refused genetic testing; thus, there were no biological samples of the fetus. WES testing of the couple revealed that the mother harbored a heterozygous variant, NM_003413: c.100_122del (p.Met35Leufs*87), in *ZIC3* on chromosome X. Considering that the male fetuses of the previous three pregnancies showed persistent arterial trunk, we speculated that the three male fetuses were highly likely to have the hemizygous variant, NM_003413: c.100_122del, in *ZIC3*^35,36^, inherited from their mother (Fig. 6).

#### Secondary/Incidental findings and carrier status for recessive diseases on pregnant couples

All 15 pregnant couples were selected to report secondary findings and carrier status for recessive diseases, of which five families were detected (Table 2). In Case 1, a pathogenic variant of *LDLR* was detected in the fetus and father, interpreted as a medically actionable secondary finding. In case 3, a likely pathogenic variant c.2254_2255dup (p.Gly753Alafs*10) in *THSD4* was detected in the fetus, and was classified as a medically actionable incidental finding, which has been reported as pathogenic in multiple unrelated patients, predisposing them to inherited thoracic aortic aneurysm^37^, additionally, a likely pathogenic variant in *FANCD2* was identified in the fetus and mother.

**Table 2:** Secondary/Incidental findings and carrier results for recessive diseases on pregnant couples.

Case ID	Associated disorders/OMIM inheritance model/	Ref Gene	Transcripts	Variant/Consequence/ GRCh37 dbSNP	ACMG classification	Origin	Significance
1	Hypercholesterolemia, familial 143890 SD	*LDLR*	NM_000527.5	c.1747C > T (p.His583Tyr) Missense variant Chr19:11227576 rs730882109	Pathogenic (PS4, PP1_Strong, PS3_Supporting)	Pat	Secondary finding Medically actionable
3	Aortic aneurysm, familial thoracic 12 619825 AD	*THSD4*	NM_024817.3	c.2254_2255dup (p.Gly753Ala fs*10) Frameshift variant Chr15:72040771	LP (PVS1, PM2_Supporting)	Mat	Incidental finding Medically actionable
13	? Spermatogenic failure 22 617706 AR	*MEIOB*	NM_001163560.3	c.47delT (p. Leu16Argfs*8) Frameshift variant Chr16:1918130	LP (PVS1, PM2_Supporting)	Pat	Carrier status
				c.683-2A > G Splicing variant Chr16:1903137	LP (PVS1, PM2_Supporting)	Mat	Carrier status

*AD* autosomal dominant, *AR* autosomal recessive, *LP* likely pathogenic, *Mat* maternal, *Pat* paternal, *SD* semidominant.

In Case 13, the fetus was affected by SIT and left ventricular hyperechoic foci; although no clinically related SNV/InDel was detected in the fetus, compound heterozygous likely pathogenic SNV, c.683-2A > G, and c.47delT (p.Leu16Argfs*8) in *MEIOB*, were detected in the mother and father, respectively, suggesting that they were carriers of ?spermatogenic failure 22 (OMIM 617706).

### Associated cardiac and extra-cardiac anomalies

Among the 29 fetuses with dextrocardia, including 27 with SIT and 2 with situs inversus partialis) (Tables 1 and S1). 31.0% (9/29) of the cases had complex cardiac malformations, with VSD (2/29), DORV (3/29) as the most frequent anomalies in this cohort. In addition, 24.1% (7/29) presented with extra-cardiac anomalies, the details of which are summarized in Tables 1 and S1.

### Perinatal outcome

The pregnancy outcomes of 29 fetuses with dextrocardia were available. Of all the fetuses with dextrocardia, 19 (65.5%) opted to continue their pregnancies, 10 (34.5%) chose to terminate.

In Case 4, a 2 cm hematoma was found at the back of the fetal head after the fetus was terminated. No new anomalies were observed in the rest newborns (Fig. 1, Tables 1 and 2).

## Discussion

It is well known that WES improves the diagnostic yield of genetic disorders, thus, it is increasingly used to evaluate fetal structural anomalies using ultrasound in prenatal settings recently^38–41^. Previous studies on the prenatal diagnosis of fetal dextrocardia have mainly focused on ultrasound diagnosis; there has been little focus on the genetic causes of dextrocardia^10,42–48^. Prenatal WES testing is best undertaken in a trio sequencing form, which facilitates the timely interpretation of the variants, thereby accelerating WES analysis and reducing its turnaround time. In our cohort, a total of 29 fetuses with dextrocardia were detected using ultrasonography, after excluding routine cytogenetic abnormalities (aneuploidies and pathogenic CNVs), 14 cases were further detected by trio-WES, and one case (Case 5) was identified by parental WES due to samples of the proband and/or the induced fetus were unavailable.

Of the 15 cases performing WES, clinically relevant variants were identified in 5/15 (33.3%) cases, with diagnostic variants involving genes *DNAH5*, *DNAH11*, *LRRC56*, *PEX10*, and *ZIC3*; thus, the improved diagnostic yield of genetic causes via WES was 33.3%, the incremental diagnostic yield could facilitate the identification of accurate genetic variants that cause fetal dextrocardia, and was crucial for effective counseling, prognosis prediction, perinatal management of fetal dextrocardia, and recurrence risk prediction in the next pregnancy.

Of the 5 diagnostic variants involving genes, *DNAH5*, *DNAH11*, and *LRRC56* were associated with PCD. PCD is a clinically heterogeneous syndrome with AR inheritance caused by ciliary defects. It is characterized by chronic sinusitis, bronchiectasis, situs abnormalities, and male infertility, with a prevalence of approximately 1 in 16,000–20,000^49^. Approximately half of the patients with PCD have SI, called KS, which is caused by defects in the function of lymph node cilia during embryonic development^50^. There are at least 30 loci associated with PCD (CILD1-30), and 28 disease-causing genes of PCD have been identified to date^51^. The variant of *DNAH5,* encoding a heavy chain of outer dynein arms, is the main genetic etiologies of 15–28% of PCD families^52,53^. Twenty-nine fetal dextrocardia cases were accompanied by SI, including 27 with SIT and two with situs inversus partialis. Of the 15 who underwent further WES, four were diagnosed with PCD types 3, 7, and 39.

A history of an undiagnosed fetus (or proband) affected by multiple or recurrent similar structural anomalies yielding normal results on cytogenetic testing in the current pregnancy was highly suggestive of a genetic cause. In addition, when such couples are referred for preconception counseling and no tissue sample is available from the affected proband or the previously aborted fetus, sequencing is recommended as the first tier for both biological couples to look for shared carrier status for AR disorders^41,54^. In our study, Cases 3, 4, and 5 had multiple adverse histories of fetuses with similar structural abnormalities, which suggested a genetic etiology. WES was recommended to identify genetic variants for those families. *ZIC3*, which encodes a highly conserved zinc-finger protein, is associated with cardiac defects^55^. In Case 5, due to the samples of the proband and aborted fetuses were not available, thus, we offered parental WES for the couple to successfully explore the shared carrier status for recessive diseases that might explain the manifestation^54^.

Owing to the complexity of WES data, the interpretation of WES results can be prenatally challenging owing to limited fetal phenotype information and the unclear the genotype-phenotypic correlation information prenatally. In our cohort, compound heterozygous variants of one pathogenic/likely pathogenic variant and one VOUS variant were detected in Cases 1 and 4 (Table 1). Considering that the clinical features (SIT, mirror-image dextrocardia, bilateral ventriculomegaly) of the fetus in Case 1 were consistent with the presentations seen in PCD type 7, with or without SI, we were able to establish a diagnosis, as c.5822G > C in *DNAH11* could be classified as likely pathogenic in this case, combining the phenotype of the fetus. In addition, Case 4 had a recurrent paternally inherited pathogenic SNV in *PEX10* in combination with a maternally inherited VOUS in the same gene, and the fetus also had recurrent biallelic compound heterozygous variants of both VOUS in *DNAH11*. Considering the recurrent similar clinical features of the fetuses (SIT, mirror-image dextrocardia, thicky NT, DORV, pulmonary stenosis, absence of pulmonary valve) in the two previous pregnancies presented, which were in accordance with the presentations seen in PCD type 7, with or without SI (OMIM 611884) and peroxisome biogenesis disorder 6A/6B (OMIM 614870/614871), the frequency of these SNVs is low in the normal reference population gene database. Thus, we established a diagnosis as it could be classified as likely pathogenic in this case due to similar fetal abnormalities; this needs to be confirmed by functional studies on VOUS variants in the future.

Studies have shown that a wide spectrum of cardiac malformations and extra-cardiac anomalies, often complex, are correlated with fetal dextrocardia^4,6,48,56^, which may have an adverse prognosis. In our cohort, 31.0% (9/29) of the fetuses with dextrocardia had complex cardiac malformations, and 24.1% (7/29) presented with extra-cardiac anomalies, and 34.5% of cases were terminated. Our data support this view, and suggest that once fetal dextrocardia is detected, a detailed ultrasound examination should be performed, especially for cardiac defects, which is consistent with our cohort.

Our study had several limitations. First, although this was a prospective study, the sample size was not large enough owing to the low incidence of fetal dextrocardia^4,6^. Second, six cases were referred from other hospitals; thus, it may not represent the true distribution of fetal dextrocardia. Third, only 15 cases underwent WES in our cohort, and 14 cases were not evaluated by WES due to the refusal of the parents at the time of diagnosis, which might bias the results of the diagnostic yield variant. Finally, the SNVs of unknown significance in Cases 1 and 4 remain to be further functionally investigated to guide the reproductive decisions of the affected families.

In conclusion, fetuses with dextrocardia are often accompanied with intracardiac and extra-cardiac malformations, especially fetuses accompanied by SI, who are at high risk of PCD. A thorough fetal structural assessment should be performed once dextrocardia is detected. Trio-WES is recommended following normal fetal karyotyping and CMA results, as it improves the diagnostic utility of genetic variants of fetal dextrocardia, accurately assesses fetal prognosis, guides their perinatal management and the reproductive choice of affected families.

### Supplementary Information

Supplementary Table S1.

## Data Availability

The sequence data supporting the results of this study has been deposited NCBI with the main entry code PRJNA1066892. Sequence data that support the findings of this study are not publicly available in order to comply with hospital and IRB policy. According to the consent form, sequencing data cannot be accessed without patient’s permission. But they are available from the corresponding author upon reasonable request.

## Abbreviations

ACMG: American college of medical genetics and genomics
AMP: Association for molecular pathology
CMA: Chromosomal microarray analysis
P/LP: Pathogenic/likely pathogenic
WES: Whole exome sequencing
TOP: Termination of pregnancy
