# Supplementary material for: Prenatal genetic diagnosis of fetuses with dextrocardia using whole exome sequencing in a tertiary center
Source: Sci Rep. 2024 Jul 15;14:16266. doi: 10.1038/s41598-024-67164-w (PMC11251054; doi:10.1038/s41598-024-67164-w)
Supplement: Supplementary file 1 — Supplementary Table S1. [file 41598_2024_67164_MOESM1_ESM.docx]

**Supplemental information**

**Table S1. The clinical information on 14 fetuses with dextrocardia who refused trio-WES testing**

| **Case ID** | **Fetal ultrasound finding** | **Gestational weeks at diagnosis** | **Maternal age** | **Pregnancy outcomes** |
| --- | --- | --- | --- | --- |
| 16 | SIT, mirror-image dextrocardia, | 15+ | 27 | LB |
| 17 | SIT, mirror-image dextrocardia, tricuspid regurgitation, bilateral ventriculomegaly | 21 | 31 | TOP |
| 18 | Dextrocardia, single ventricle, pulmonary stenosis, DSVA | 17 | 28 | TOP |
| 19 | SIT, mirror-image dextrocardia | 24 | 38 | LB, normal after hernia repair surgery |
| 20 | SIT, mirror-image dextrocardia, bilateral kidney enlargement and enhanced parenchymal echo | 17+ | 26 | TOP |
| 21 | SIT, mirror-image dextrocardia | 25 | 34 | LB |
| 22 | SIT, mirror-image dextrocardia | 18 | 29 | LB |
| 23 | SIT, mirror-image dextrocardia, intra-cardiac echogenic foci | 22+ | 33 | LB |
| 24 | SIT, mirror-image dextrocardia, fetal endocardial pad defect, single major artery | 16+ | 31 | TOP |
| 25 | SIT, mirror-image dextrocardia | 20+ | 22 | LB |
| 26 | SIT, dextrocardia，polyhydramnios | 23 | 35 | LB |
| 27 | SIT, mirror-image dextrocardia, left aortic arch, right ductus arteriosus, ventricular septal defect, bilateral choroid plexus cyst | 21 | 32 | TOP |
| 28 | SIT, mirror-image dextrocardia | 14 | 25 | LB |
| 29 | SIT, mirror-image dextrocardia | 23+ | 33 | TOP |

**Abbreviations:** DSVA, double superior vena cava; LB, live birth; SIT, situs inversus totalis; TOP, termination of pregnancy; WES, whole exome sequencing
